# Participation in leisure activities and quality of life of people with psychosis in England: a multi-site cross-sectional study

**DOI:** 10.1186/s12991-023-00438-1

**Published:** 2023-03-13

**Authors:** Kayonda Hubert Ngamaba, Martin Webber, Penny Xanthopoulou, Agnes Chevalier, Domenico Giacco

**Affiliations:** 1 International Centre for Mental Health Social Research (ICMHSR), School for Business and Society, University of York, Heslington, York, YO10 5DD UK; 2 Mental Health Research Group, College of Medicine and Health, University of Exeter, College House (1.05), St. Luke’s Campus, Exeter, EX1 2LU Devon UK; 3 Unit for Social and Community Psychiatry, (WHO Collaborating Centre for Mental Health Service Development), Barts and the London School of Medicine, Newham Centre for Mental Health, Queen Mary University of London, London, E13 8SP UK; 4 Division of Health Sciences, Warwick Medical School, University of Warwick, Coventry, CV4 7AL UK

**Keywords:** Quality of life, Diagnosis of psychosis, Schizophrenia, Leisure activities, Mental health

## Abstract

**Background:**

Leisure activities can improve quality of life in the general population. For people with psychosis, negative symptoms (e.g. being unmotivated, difficulty in sticking with activities) are often a barrier to engaging in social leisure activities. However, we do not know if participation in leisure activities is associated with quality of life in this group and, whether psychosocial interventions should aim to increase leisure activities.

**Aim:**

This study investigates participation in social leisure activities of people with psychosis and whether their participation is associated with better quality of life.

**Methods:**

A cross-sectional survey was conducted in 6 NHS mental health trusts. Adults aged 18–65 (*N* = 533) with a diagnosis of a psychosis-related condition (ICD-10 F20-29) were recruited from outpatient secondary mental health services. Several measures were used including an adapted version of the Time Use Survey (TUS), the Social contacts assessment (SCA) and Manchester Short Assessment of Quality of Life (MANSA). A Structural Equation Model (SEM) was used to explore the relationships between participation in leisure activities and quality of life, and whether social contacts mediated the link.

**Results:**

Participants attended an average of 2.42 (SD = 1.47) leisure activities in the last 7 days. Their quality of life increased with the number of leisure activities they attended. Participation in leisure activities was positively associated with quality of life in people with psychosis (*B* = 0.104, SE = 0.051, *p* = 0.042, 95% CI [0.003 to 0.204]). Leisure activities predicted social contacts, but the link between social contacts and the quality of life was not significant. After controlling for sociodemographic factors, being female and unemployed were negatively linked with quality of life (*B* = − 0.101, SE = 0.048, *p* = 0.036, 95% CI [− 0.196 to − 0.006; *B* = − 0.207, SE = 0.050, *p* = 0.001, 95% CI [− 0.305 to − 0.108, respectively].

**Conclusion:**

People with psychosis who attend more leisure activities have a higher quality of life. Quality of life was lower amongst female and unemployed participants who attended leisure activities. Intervention which helps improve participation in leisure activities may be beneficial for people with psychosis.

*Trial registration* number ISRCTN15815862.

**Supplementary Information:**

The online version contains supplementary material available at 10.1186/s12991-023-00438-1.

## Background

Leisure activities have been associated with quality of life [1]. Engaging in leisure activities is associated with improvements in personal relationships, self-esteem, time structure and the creation of capital networks and employment [2, 3].

The government in England is promoting the quality of life of primary care patients through sport and leisure activities. For example, in 2019, it launched a new National Academy for Social Prescribing (NASP) (see www.socialprescribingacademy.org.uk/) to give every patient in the country access to sport and leisure activities [4, 5].

Previous studies have reported that participation in leisure activities can positively affect the quality of life of older people [6] and people with common mental health conditions [3, 7–9].

A 6 year panel study conducted amongst adults aged 50–59 in Japan found that involvement in both leisure activities, such as ’hobbies or cultural activities’ and ’exercise or sports’, was significantly and positively related to mental health status in both men and women [10]. Nevertheless, in men, both ’hobbies or cultural activities’ and ’exercise or sports’ were significantly related to mental health status only when conducted ’with others’. In women, the effects of ’hobbies or cultural activities on mental health status were not different regardless of the ways of participating, whilst the result of ’exercise or sports’ was the same as that in men [10]. Moreover, a prospective cohort study conducted in the western part of Denmark amongst 15–24 years old found that boys are more physically active than girls [9].

However, most of these studies have investigated the link between physical leisure activities (e.g. exercise, gym and sports) and quality of life in people with common mental health conditions, such as anxiety and depression [3, 7], in unemployed people [3], people with cancer [8], or people with poor mental health in early adulthood [9]. There is no data as to whether participation in leisure activities has an effect on the quality of life of patients with psychotic disorders.

Leisure activities refer to any un-obligated time or activity that brings direct satisfaction, a state of being content and happy following participation in specific activities performed in your own spare time without any pressure for survival [2, 6, 11, 12]. This includes activities, such as going to the cinema, going to an event as a spectator, going to a museum, library, shopping centre, or going to an entertainment, outdoor trips, or going to a day centre/community group, attending a religious group/activity, or being visited by friends or visiting friends or going out for a meal. Participation in leisure activities could be a complex procedure for some people because it includes the processes of finding, planning and implementing appropriate and interesting leisure activities [13]. Despite several studies suggesting that leisure activities contribute to the quality of life in the general population, people with psychosis might experience problems with engaging in leisure activities due to negative symptoms [14]. Previous studies have found that anhedonia, emotional blunting and low energy affect motivation and ability to engage, establish and maintain social relationships [15, 16]. Second, people with psychosis might experience social disadvantages such as unemployment or living alone with fewer opportunities to use social skills [17, 18]. Third, passive social withdrawal, which is a core behavioural feature in schizophrenia, is associated mainly with asociality, though it can also be seen secondary to psychotic symptoms. While one person may lack a desire for affiliation, another may be isolated because of paranoid fears [19].

This study aims to investigate whether attendance in social leisure activities is associated with the quality of life of people with psychosis in England. It addresses two evidence gaps. First, little is currently known about the participation in leisure activities of people with psychosis. Second, there have been no multi-site cross-sectional studies exploring the association of leisure activities with the quality of life of patients with psychotic disorders. The findings of this study may help to inform intervention development which may support the engagement of people with psychosis in leisure activities.

## Methods

### Study design and participants

A cross-sectional survey was conducted in NHS Community Mental Health Team (CMHT)s in England. CMHT is an umbrella term used to describe a multi-professional team involved in the delivery of mental health care and it’s formed of community psychiatric nurses, occupational therapists, social workers, psychologists, psychiatrists and health care support workers.

From June 2017 to May 2018, participants were recruited in six participating NHS Trusts covering a range of geographical areas, in both urban and rural contexts: Cornwall Partnership NHS Foundation Trust; Devon Partnership NHS Trust; East London NHS Foundation Trust (covering East London, Luton and Bedfordshire); Oxford Health NHS Foundation Trust (covering large areas of Oxfordshire and Buckinghamshire) and Somerset Partnership NHS Foundation Trust; Tees, Esk and Wear Valleys NHS Foundation Trust (covering County Durham, Darlington, Teeside, York and North Yorkshire).

Participants were identified by clinicians or clinical study officers from CMHT caseloads. Whilst CMHTs look after patients with different diagnoses, this study focussed on out-patients with a diagnosis of a psychotic disorder according to the International Classification of Disease-10 (ICD-10) codes F20-29.

Participants were eligible for inclusion if they met the following criteria:

•Adults aged 18–69 years old.
•A clinical diagnosis of a psychotic disorder according to the International Classification of Disease-10 (ICD-10) codes F20-29, as identified in clinical records.
•Receiving care from outpatient secondary mental health services or primary care services.
•Have the capacity to provide informed consent.
•Able to communicate in English.

Exclusion criteria:

•A current and primary diagnosis of substance use disorder (ICD-10 F10-19).
•Had been hospitalised in the previous week (although these potential participants could be re-approached at a later time).
•Their postcodes could not be obtained because they were homeless or living in temporary accommodation at the time of the survey.

### Procedures and measures

Eligible participants were identified by clinicians or clinical study officers and asked for their agreement to speak to a member of the research team. Participants then completed the study questionnaires and researchers accessed participant clinical records to retrieve clinical and sociodemographic characteristics. All participants who agreed to take part in this study were interviewed in quiet rooms in community mental health teams, primary care settings, or at participant’s homes using standardised case report forms. All interviews were face-to-face and took about 45 min to complete. Several measures were used during the assessments:

First, the UK Time Use Survey (TUS) [20] as adapted by Priebe et al. [21] to focus on activities outside of home (See Additional file 1: Table S1 Time Use Survey Leisure Activities), was used to assess participation in leisure activities lasting more than 10 min during the previous week. The adapted TUS was selected for its focus on social leisure activities which take place outside the home and include the following: Been to a museum/art gallery, Been to a place of entertainment (e.g. dance, club, bingo and casino), Been to an event as a spectator (e.g. sports event, theatre and live performance), Been to outdoor trips (e.g. picnics, beach), Been to a library, Been to a community social group/day centre, Been to a shopping centre, Been to the cinema, Attended a religious group/activity/service, Been visited by friends, Visited friends and Been out to eat/drink at a café/restaurant/pub. If they participated in an activity that was not on the list, they were also asked to specify the activity they completed. For each activity they had completed, participants were asked to report (i) the number of times they completed the activity (i.e. only taking short breaks in between constituted one activity), (ii) the duration to the nearest 10 min, (iii) whether participation took place alone or with someone else, (iv) and if with someone else, to define their relationship to this individual: parent, sibling, friend, partner or other. Participants were asked not to double-count time spent in activities (e.g. going out for a meal and visiting a friend) but select the activity which best describes the event.

Second, the number of self-reported social contacts in the last 7 days was measured with the Social Contact Assessment (SCA) [22]. The instrument asks participants to list the initials of social contacts with who they have been in contact in the last 7 days to generate a total number of social contacts. For ‘being in contact’, we mean that they can name them and have had a chat (more than just greetings) in the last week. Participants were asked not to include people they were living with, health professionals or people they worked with, unless their contact that took place outside of and was unrelated to work. For each contact, participants were asked to define the type of relationship (1 = parent, 2 = sibling, 3 = friend, 4 = partner, 5 = other), on how many days in the last week they had been in face-to-face contact, whether the meeting was one-to-one, in a group or both, on how many days they used voice or video call, mail or text message, whether they can talk to them about their personal feelings/worries and whether they did something for them and vice-versa.

Third, participants reported satisfaction with different aspects of their life using the twelve items of the Manchester Short Assessment of Quality of Life (MANSA), rated on a scale from 1 (very dissatisfied) to 7 (very satisfied). Participants were asked to rate their satisfaction with: life as a whole, job situation, financial situation, friendships, leisure activities, accommodation, personal safety, the people they live with, sex life, family relationships physical health and mental health. MANSA has been widely used to assess the quality of life of people with psychosis and its psychometric properties have been well established [23–25].

Researchers collected additional participant characteristics, such as age, gender (male/female), marital status (single/in a relationship), country of birth (born in the United Kingdom/born in a different country), an education level (primary/secondary/further), living situation (living alone/living with someone), accommodation (living independently/living in supported accommodation), employment (employed/not employed), receipt of welfare benefits (or not) and length of illness (calculated in the number of years from the day of the first contact with mental health services). These were collected from participants’ assessments and checked against available data in medical records.

### Description of the sample

**Fig. 1:**
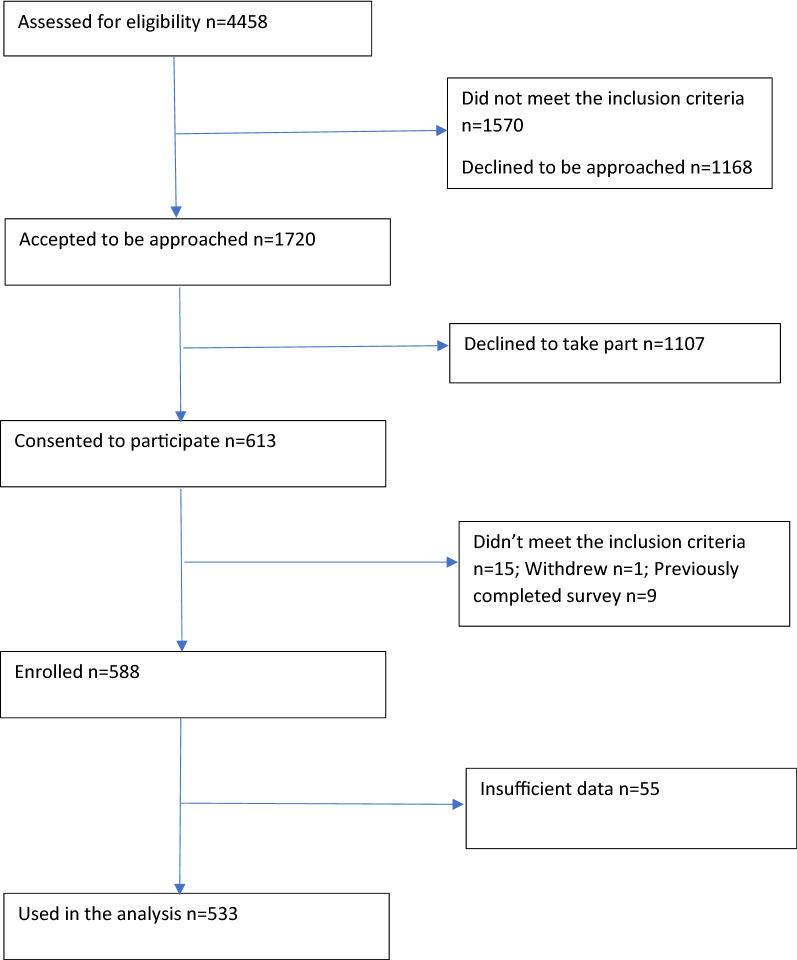
Flow diagram describing recruitment of community participants with psychosis

Figure 1 presents the flow of participant recruitment (see Fig. 1). Once inclusion criteria were applied, 2888 were eligible. Of those, we were able to contact and explain the study to 1720 people of whom 613 agreed to take part representing a consent rate of 35%. For a small number of consented individuals, it only became apparent that they did not meet certain eligibility criteria (e.g. capacity to provide informed consent, been hospitalised in the previous week) upon meeting.

A sample of 588 participants enrolled in the study, of whom 533 reported their participation in leisure activities during the previous week. The majority of participants (*N* = 407) responded to all 12 questions related to quality of life with the MANSA. All analyses involving the outcome variable (i.e. quality of life) used the final sample (*N* = 407). The missing data was handled and the list-wise deletion was used because data was missing completely at random [26].

### Ethics committee approval

The West Midlands—Solihull Research Ethics Committee (17/WM/0191) approved the study. All participants were given the information sheet and written informed consent was obtained for all participants.

### Statistical analysis

Descriptive statistics (i.e. number of participants and percentage) for socio-demographic factors are reported. Mean and Standard Deviation are reported for quality of life, leisure activities and social contacts (see Table 1). Quality of life is the mean score (on scale of 1 to 7) of the 12 items on the MANSA. Leisure activities are the number of activities participants attended in the last 7 days. Social contacts are the total number of face-to-face social contacts with different individuals in the last 7 days.

**Table 1:** Descriptive statistics: socio-demographic information

Variables	*N* = 533 (%)
Gender *N* (%)	
Female	184 (34.5%)
Male	348 (65.2%)
Prefer not to say	1 (0.2%)
Age	
Age 20 to 39	198 (37.1%)
Age 40 to 59	300 (56.2%)
Age 60 to 69	35 (6.5%)
Marital status N (%)	
Single	402 (75.4%)
Married	65 (12.2%)
Co-habiting/civil partnership	13 (2.4%)
Separated	6 (1.1%)
Divorced	37 (6.9%)
Widow/Widower	7 (1.3%)
Not known/Missing	3 (0.5%)
Education N (%)	
Primary education or less	40 (7.5%)
Secondary education	223 (41.8%)
Tertiary/Further Education	233 (43.7%)
Other general Education	24 (4.5%)
Unknown/Missing	13 (2.4%)
Accommodation *N* (%)	
Independent accommodation	392 (73.5%)
Supported accommodation	114 (21.3%)
Homeless/Roofless	6 (1.1%)
Other accommodation	19 (3.5%)
Living condition *N* (%)	
Living alone	246 (46.1%)
Living with a partner or family	186 (34.9%)
Living with friend(s)	8 (1.5%)
Living in shared accommodation	90 (16.8%)
Employment *N* (%)	
Full-time paid or self-employed	20 (3.7%)
Part-time paid or self-employed	29 (5.4%)
Voluntary employ	57 (10.6%)
Unemployment	375 (70.3%)
Student	19 (3.5%)
Housewife/husband	5 (0.9%)
Retired	8 (1.6%)
Other	10 (1.8%)
State benefits *N* (%)	
No	43 (8.0%)
Yes	473 (88.7)
Main psychiatric diagnosis *N* (%)	
Schizophrenia	250 (68.5%)
Schizotypal disorder	3 (0.6%)
Delusional disorder	12 (2.3%)
Brief psychotic disorder	13 (2.6%)
Schizoaffective disorder	81 (15.8%)
Psychosis NOS	31 (6.1%)
Ethnicity *N* (%)	
White British	340 (63.7%)
Black/Black British	69 (12.9%)
Asian/Asian British	61 (11.4%)
Score in the last 7 days	Mean (SD)
Quality of life MANSA score	4.97 (1.01)
Leisure activities attended	2.42 (1.47)
Social contacts made	2.86 (2,62)

*MANSA* Manchester Short Assessment of Quality of Life, *SD* Standard Deviation

### Structural equation model (SEM)

Three basic concepts are commonly used by researchers for SEM reporting: (1) a well-developed theoretical model, (2) operational definitions of the observed/unobserved variables and (3) graphical representation of the theorised model. A checklist for SEM reporting is used [26, 27].

SEM has been widely used as a series of statistical methods because it allows analyses, evaluation and interpretation of complex relationships between one or more independent variables and one or more dependent variables [28, 29].

**Fig. 2:**
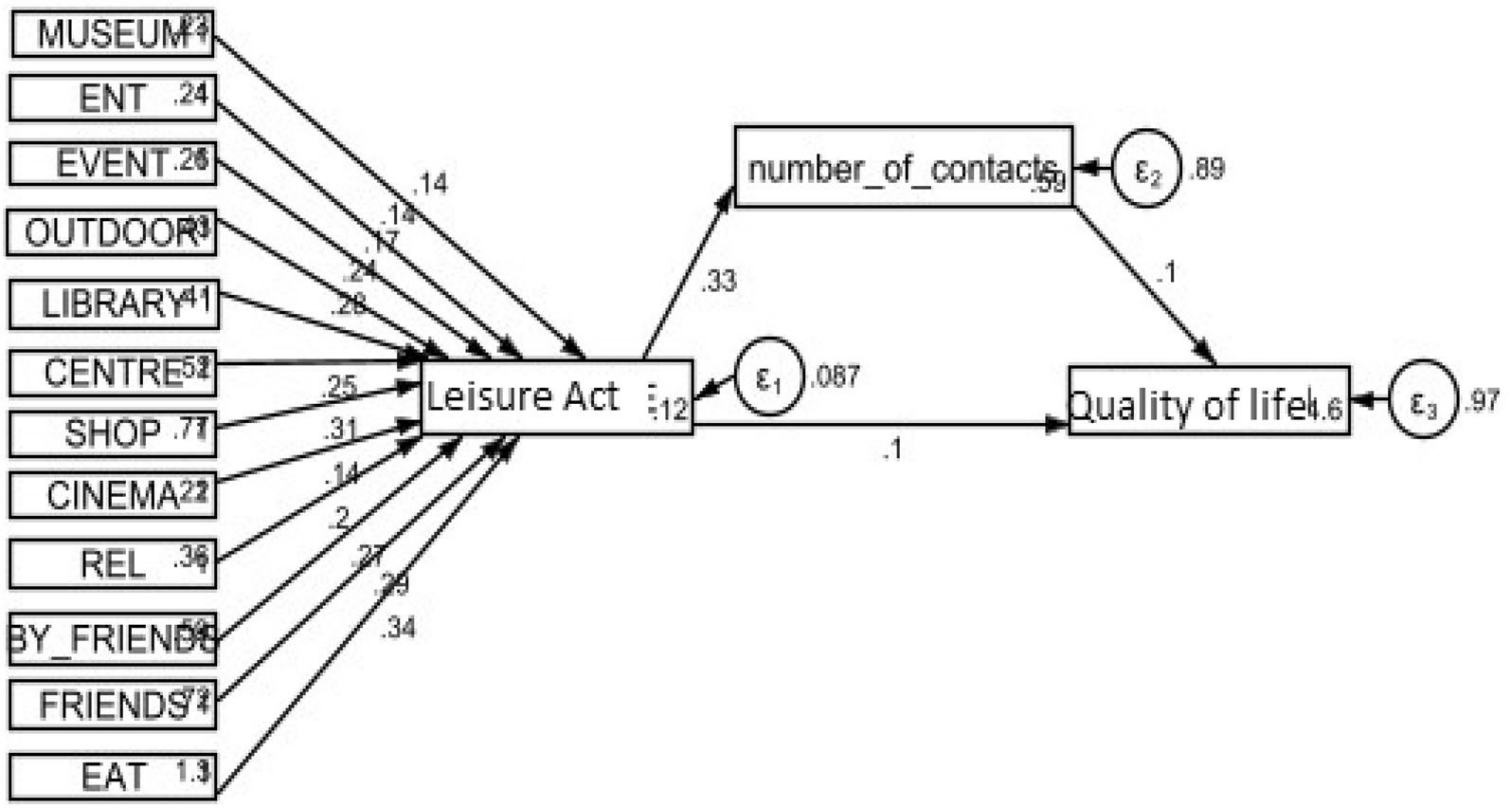
Relationship between the Participation in leisure activities and the Quality of life. The number of social contacts is mediated the association. Standardised coefficients and values

**Fig. 3:**
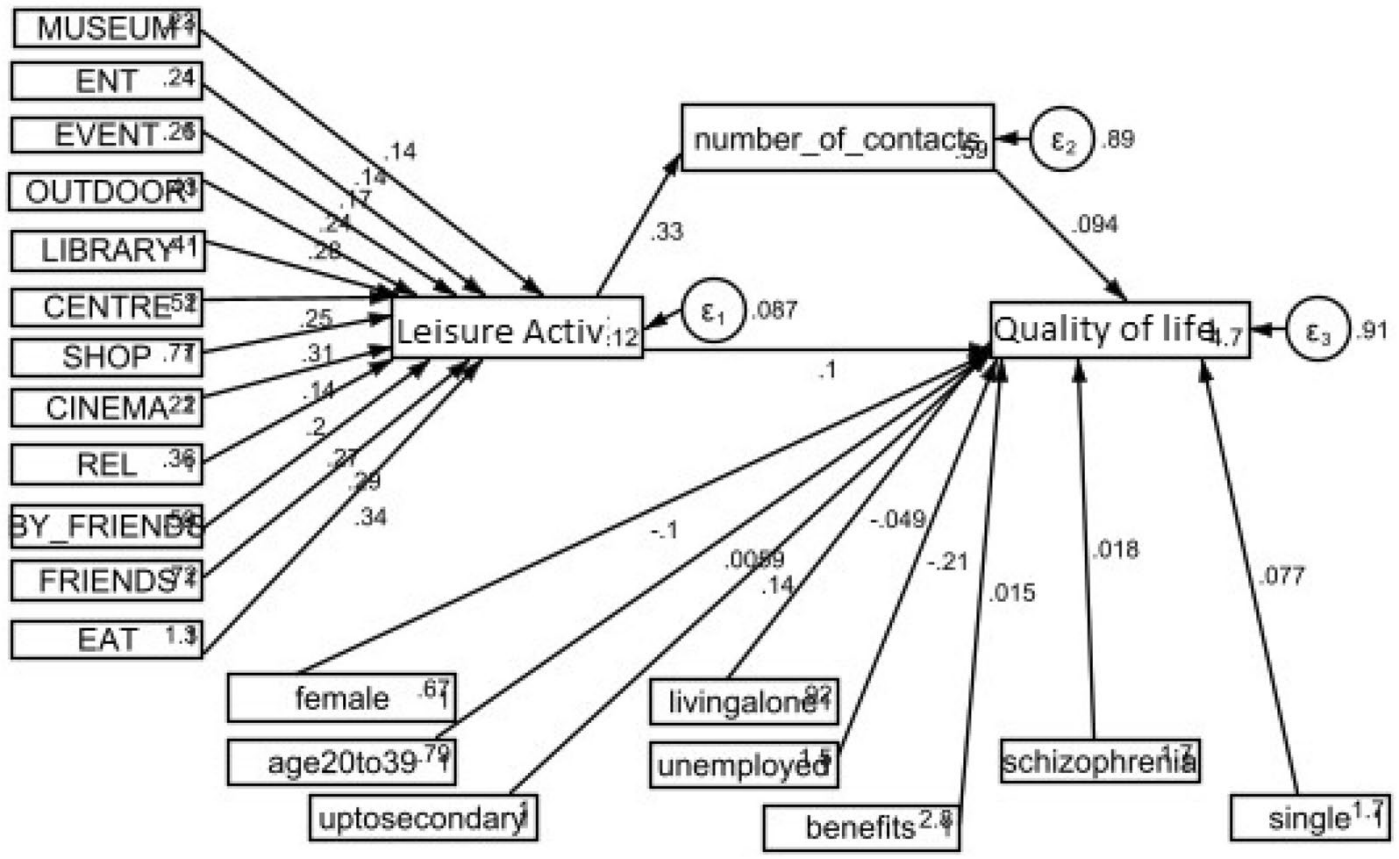
Relationship between the Participation in leisure activities and the Quality of life, after controlling for socio-demographic and other factors such as diagnosis. Standardised coefficients and values

SEM has observed variables, also called measured variables that are represented by a square or rectangle in the traditional graphic. The unobserved variables, also termed latent variables are represented by large circles, and the small circles represent the measurement errors in the observed variables. Two other terms associated with SEM are exogenous, similar to independent variables, and endogenous, similar to dependent/outcome variables. Single-head arrows (paths) represent directional effects from 1 variable (latent or observed) to another. In our structural model, we used the path analysis where leisure activities, social contacts and quality of life were measured (see Figs. 2 and 3). In the diagram, the regression coefficient, the direction and the magnitude of the relationship, is set at 1 for each path [26, 27].

We explored the relationships between participation in leisure activities and the quality of life through the proposed SEM. This study hypothesises that participation in leisure activities has a positive influence on quality of life. In addition, it is hypothesised that social contacts are thought to have mediating effects between participation in leisure activities and quality of life. This reflects the general logic model of the study ‘social engagement—> social contacts—> quality of life’ [30]. In other words, in our model, participation in leisure activities is an independent variable, social contacts are a mediator and quality of life is a dependent variable. The fit of the model is evaluated using SEM. The relationships are graphically presented in Figs. 2 and 3.

We conducted the statistical analyses in two steps. The first step looked at the relationship between participation in leisure activities, social contacts and quality of life without involving socio-demographic factors (see Fig. 2 and Table 2). The second step was built from step one by adding into the model all socio-demographic factors and other factors such diagnosis (see Fig. 3 and Table 3).

**Table 2:** Correlations, means and standard deviations (SD) for individual items (quality of life score, leisure activities attended and social contacts made)

	Quality of life	Leisure activities	Social contacts	Means	SD
Quality of life (*N* = 407)	1.000			4.97	1.01
Leisure activities (*N* = 535)	0.138^b^	1.000		2.42	1.47
Social contacts (*N* = 535)	0.137^b^	0.334^a^	1.000	2.86	2.62

*ns* non-significant

^a^p < 0.001

^b^p < 0.01

^c^p < 0.05

**Table 3:** SEM investigating the association between the participation in leisure activities and quality of life; the mediating effect between social contacts and quality of life

Endogenous variables	Exploratory variables	Estimates (coefficient)	Std Err	P-value	[95%	CI]
Leisure activities	Museum	0.14^a^	0.02	0.001	0.11	0.17
	Entertainment	0.14^a^	0.02	0.001	0.11	0.17
	Event	0.17^a^	0.02	0.001	0.14	0.20
	Outdoor	0.24^a^	0.01	0.001	0.21	0.27
	Library	0.28^a^	0.01	0.001	0.25	0.31
	Centre	0.25^a^	0.01	0.001	0.23	0.28
	Shopping	0.31^a^	0.01	0.001	0.28	0.33
	Cinema	0.14^a^	0.02	0.001	0.12	0.17
	Religious act	0.20^a^	0.01	0.001	0.17	0.23
	Visit by friend	0.27^a^	0.02	0.001	0.24	0.30
	Visit friends	0.29^a^	0.02	0.001	0.26	0.32
	Out to eat	0.34^a^	0.01	0.001	0.31	0.36
	_constant	0.12^a^	0.03	0.001	0.06	0.18
Social contacts	Leisure activities	0.33^a^	0.04	0.001	0.24	0.41
	_cons	0.59^a^	0.10	0.001	0.39	0.79
Quality of life	Leisure activities	0.10^c^	0.05	0.04	0.00	0.20
	Social contacts	0.10^c^	0.05	0.05	0.00	0.20
	_constant	4.62^a^	0.21	0.001	4.22	5.03

N = 407. Used Structural equation model (SEM); Standardised coefficients and values. All variables in the model were observed. Endogenous variables (variables that were influenced by other variables in our model): Participation in leisure activities, Number of social contacts and Quality of life. Exogenous variables (variables that were not influenced by other variables in our model): Being to the Museum, Entertainment (Club), Event (Sport or Music), Outdoor Trips, Library, Community Centre, Shopping, Cinema, Religious Activities, Been Visited by Friends, Visited Friends and Been out to Eat

*ns* non-significant, *95% CI* 95% Confidence Interval, *LR* test of model vs. saturated: chi^2^ (24) = 43.67 Prob > chi2 = 0.0083

^a^P-value significant: 0.001

^b^p < 0.01

^c^p < 0.05

Dummy variables were created for socio-economic factors to investigate the association of some variables of interest with the quality of life. For example, whether there is gender difference in the way male and female participate in leisure activities [10]; whether being unemployed [17], single [10], living alone or having a specific diagnosis [14, 19] influence the quality of life of people with psychosis. Dummy variables created (e.g. 1 = female and 0 = otherwise; 1 = single and 0 = otherwise; 1 = young adults and 0 = otherwise; 1 = up to secondary education and 0 = otherwise; 1 = independent accommodation and 0 = otherwise; 1 = living alone and 0 = otherwise; 1 = unemployed and 0 = otherwise; 1 = on benefits and otherwise; 1 = schizophrenia and 0 = other diagnoses).

We reported odds ratios (OR) with their corresponding 95% confidence intervals (CI) and *P*-value significant of 0.05. The final sample size was reported and we explained how missing data was handled. For example, the list-wise deletion was used because data was missing completely at random [26].

We also checked whether the quality of life and other factors, such as age and education, were normally distributed (See Additional file 2: Distribution Graphs). All statistical analyses were conducted using Stata 17.0 [31].

## Results

### Participant characteristics

The majority of participants were male, single, middle-aged and living alone (not with parents or friends). Most participants had completed further education and were unemployed, on state support benefits and living in unsupervised accommodation (i.e. independent accommodation). They were mostly white, and diagnosed with schizophrenia (see Table 1).

The mean quality of life score was 4.97 on a scale of 1 to 7 (see Table 1). On average, participants had attended 2.42 leisure activities in the last week. The quality of life scores increased slightly with the number of leisure activities participants had attended. For example, from zero to 1 leisure activity, the quality of life mean score increased from 4.67 (SD = 1.11) to 4.89 (SD = 1.02), and then with 2 leisure activities, the quality of life mean score increased to 5.02 (SD = 0.88). From 2 to 3 leisure activities, the quality of life decreased to 4.81 (SD = 0.99). From 4 to 5 leisure activities, the quality of life mean score increased from 5.15 (SD = 0.95) to 5.33 (1.07). The most frequent leisure activities were “going out to eat” or “shopping for leisure”. On average, participants had 2.86 social contacts in the last week (see Table 1).

### Overall SEM model fit

The theoretical model of the factors involved in quality of life proposed in this study met the preliminary fit criteria and overall model fit [28]. For example, the correlation coefficients of the observed variables, means and standard deviations have been checked and reported [26, 27]. We found that the correlations between leisure activities attended and social contacts made was significant and positive (*r* = 0.334, *p* < 0.01). We had a positive and significant correlation between quality of life and leisure activities (*r* = 0.138, *p* < 0.01) (see Table 2). Also, none of the standardised error variance estimates were negative (see Table 3). The standardised error variance was smaller between 0.014 and 0.051 (*p* < 0.05). The chi-square of the overall model fit between the theoretical model and the data was 43.67, *p* = 0.008. Other indices, such as the goodness-of-fit index root mean squared error of approximation (RMSEA) was 0.045. Importantly, a Checklist for SEM Model has been used and the main results for the structural model and the mediating effect have been reported [27] (see Tables 3 and 4).

**Table 4:** SEM investigating the association between the Participation in leisure activities and quality of life; the mediating effect between social contacts and quality of life; After controlling for socio-demographic factors

Endogenous variables	Exploratory variables	Estimates (coefficient)	Std. err	P-value	[95%	CI]
Leisure activities	Museum	0.14^a^	0.01	0.001	0.11	0.16
	Entertainment	0.13^a^	0.01	0.001	0.10	0.16
	Event	0.16^a^	0.01	0.001	0.13	0.19
	Outdoor	0.23^a^	0.01	0.001	0.20	0.26
	Library	0.27^a^	0.01	0.001	0.25	0.30
	Centre	0.25^a^	0.01	0.001	0.22	0.28
	Shopping	0.30^a^	0.01	0.001	0.27	0.33
	Cinema	0.14^a^	0.01	0.001	0.11	0.17
	Religious act	0.20^a^	0.01	0.001	0.17	0.23
	Visit by friend	0.27^a^	0.02	0.001	0.24	0.30
	Visit friends	0.28^a^	0.01	0.001	0.26	0.32
	Out to eat	0.33^a^	0.01	0.001	0.31	0.36
	_constant	0.11^a^	0.03	0.001	0.06	0.18
Social contacts				0.001		
	Leisure activities	0.32^a^	0.04	0.001	0.24	0.41
	_constant	0.58^a^	0.10	0.001	0.39	0.79
Quality of life						
	Leisure activities	0.101^c^	0.05	0.047	0.00	0.20
	Social contacts	0.09 ns	0.05	0.069	− 0.01	0.20
	Female	− 0.10^c^	0.05	0.036	− 0.20	− 0.01
	Age20to39	0.01 ns	0.05	0.907	− 0.09	0.10
	Education up to secondary	0.14^b^	0.05	0.004	0.05	0.24
	Single (not married)	0.07 ns	0.05	0.120	− 0.02	0.17
	Living alone	− 0.04 ns	0.05	0.341	− 0.15	0.05
	Unemployed	− 0.20^a^	0.05	0.001	− 0.31	− 0.11
	On state benefits	0.01 ns	0.05	0.778	− 0.09	0.12
	Diagnosis of schizophrenia	0.01 ns	0.05	0.715	− 0.08	0.11
	_constant	4.72^a^	0.29	0.001	4.15	5.30

N = 407. Used Structural equation model (SEM); Standardised coefficients and values. All variables in the model were observed. Endogenous variables (variables that were influenced by other variables in our model): Participation in leisure activities, Number of social contacts and Quality of life. Exogenous variables (variables that were not influenced by other variables in our model): Being to the Museum, Entertainment (Club), Event (Sport or Music), Outdoor Trips, Library, Community Centre, Shopping, Cinema, Religious Activities, Been Visited by Friends, Visited Friends and Been out to Eat, Being Female, Being young adult age 20 to 39, Having an Education up to secondary, Being single (not married), Living alone (accommodation), Being unemployed, Receiving state benefits and Having a diagnosis of schizophrenia

*ns* non-significant, *95% CI* 95% Confidence Interval, *LR* test of model vs. saturated: chi^2^ (40) = 90.39 Prob > chi2 = 0.0000

^a^P-value significant: 0.001

^b^p < 0.01

^c^p < 0.05

### Effects on quality of life in the SEM theoretical model

In our theoretical model of quality of life, this study treated participation in leisure activities as independent variables, social contacts as a mediator and quality of life as a dependent variable.

The model presented in Fig. 2 shows a positive relationship between the participation in leisure activities and the quality of life (*B* = 0.104, *p* = 0.042). Participation in leisure activities was positively linked with social contacts (*B* = 0.326, *p* = 0.001) that were positively associated with the quality of life (*B* = 0.103, *p* = 0.045). People who participated to more leisure activities were likely to have more social contacts and to have higher quality of life.

When socio-demographic factors were included into the model, the direct effects of participation in leisure activities on quality of life remained positive *B* = 0.101, *p* = 0.047 (see Fig. 3 and Table 4). The relationship between leisure activities and social contacts also remained positive *B* = 0.326, *p* = 0.001. In contrast, social contacts were no longer associated with quality of life *B* = 0.094, *p* = 0.069 (see Fig. 3 and Table 4).

Several social demographic factors were associated with the quality of life. Being female and unemployed were negatively linked with the quality of life (*B* = − 0.101, *p* = 0.036; *B* = − 0.207, *p* = 0.001, respectively). Only completing education up to secondary school was positively associated with the quality of life, *B* = 0.141, *p* = 0.004. No other sociodemographic factors were associated with quality of life (see Table 4).

## Discussion

This study investigated whether participation in social leisure activities is associated with the quality of life of people with psychosis in England. The study found that participation in leisure activities was positively associated with the quality of life of people with psychosis. On average, participants attended two leisure activities in the last week and their quality of life increased slightly with the number of leisure activities attended. The mean quality of life score of 4.97 (SD = 1.01) found in the present study is slightly higher to that found by Priebe and colleagues of 4.56 (SD = 0.51) in participants with a diagnosis of a psychotic disorder [25].

Our findings suggest that, whilst people with psychosis may have specific difficulties in engaging with social activities [22, 32, 33], weekly leisure activities need to be encouraged because there are associated with a higher quality of life.

Previous studies which report a positive link between the quality of life and leisure activities have suggested that this could be because leisure activities refer to any un-obligated time [12] and can increase individuals’ perception of spending their time effectively [3]. This may suggest that interventions that support people to engage in leisure activities should be used with people with psychosis. However, our model testing social contacts as a mediator found that people who participated to more leisure activities were likely to have more social contacts but not all social contacts that were beneficial to participants’ quality of life.

Our findings are in line with previous studies suggesting that participation in leisure activities is positively associated with the quality of life of vulnerable people including older people [6], and people with common mental health conditions [7–9].

This study found that both males and females participated in leisure activities. However, the link between leisure activities and quality of life was much stronger amongst males and negative amongst females. Previous studies suggest that whilst both males and females with mental health conditions can be involved in leisure activities, when it comes to the impact leisure activities have on their quality of life, males seem to benefit more from leisure activities than women [9, 10]. Whilst it is possible, that men benefit more from engaging in leisure activities than women, the gender differences in the present study need to be treated with caution because of the unbalanced sample between males and females and the small sample of female participants.

## Strengths and limitations

This study has several strengths. First, this is the first multi-site cross-sectional study investigating the link between attendance in leisure activities and the quality of life of people with psychosis in England.

Second, this study recruited a large sample across several mental health providers covering a variety of urban and rural areas in England. And finally, it may inform researchers who are developing the implementation of psychosocial interventions for people with psychosis [16, 34].

Nevertheless, it is important to recognise some limitations. First, participants were identified by clinicians or clinical study officers and asked for their agreement to take part in this research study. It is possible that people who agreed to participate were more comfortable with engagement in leisure activities compared to those who declined to take part in this study. Second, of 533 participants, 184 (34.5%) were female and 348 (65.2%) were male. This may explain why attendance in leisure activities was associated with a higher quality of life amongst male participants and lower quality of life amongst females. Third, nearly all of the people who took part in this study were taking psychotropic medications, and this study did not investigate the impact that psychotropic drugs may have on engagement in leisure activities, which in turn may affect their quality of life. Finally, this is a cross-sectional study looking at the association between attendance in leisure activities and the quality of life of people with psychosis. To establish a causal association, longitudinal or intervention studies are required to explore whether there is a change in the quality of life of people with psychosis resulting from engagement in leisure activities. It is possible that those with a higher quality of life feel better in general and are more able to engage in leisure activities.

## Conclusions

Participation in leisure activities is linked with a higher quality of life amongst people with psychosis. This study found a gender difference between males and females. The association between attendance in leisure activities and quality of life is significantly positive amongst males; and negative amongst females and the unemployed who attended leisure activities. The positive link between leisure activities and the quality of life of males with psychosis may encourage the government in England with their policy of promoting the quality of life of mental health patients through leisure activities. Further research is needed to determine whether interventions to increase attendance in leisure activities should be tried in people with psychosis.

## Supplementary Information

**Additional file 1: Table S1.** Time Use Survey Leisure Activities.

**Additional file 2.** Distribution Graphs.

## Data Availability

The datasets used and/or analysed during the current study are available from the corresponding author on reasonable request.

## Abbreviations

MANSA: Manchester short assessment of quality of life
TUS: Time use survey
SCA: Social contacts assessment
SEM: Structural equation model
NASP: National academy for social prescribing
CMHT: Community mental health team
NHS: The National Health Service
