# Supplementary material for: Participation in leisure activities and quality of life of people with psychosis in England: a multi-site cross-sectional study
Source: Ann Gen Psychiatry. 2023 Mar 13;22:8. doi: 10.1186/s12991-023-00438-1 (PMC10009983; doi:10.1186/s12991-023-00438-1)
Supplement: Supplementary file 1 — Additional file 1: Table S1. Time Use Survey Leisure Activities. [file 12991_2023_438_MOESM1_ESM.docx]

**Table S1 Time Use Survey Leisure Activities**

*I am now going to ask some questions about things that some people do in their spare time. This excludes time spent with your mental health care team. For each activity that I mention could you please tell me whether or not you have done this in the last WEEK, AND how often?*

| Activity | Number of times | Total amount of time (to the nearest 10 Minutes) | Did you do this with someone? If so who was it and what is their relationship to you? |
| --- | --- | --- | --- |
| Been to the cinema |  |  |  |
| Been to an event as a spectator (e.g., sports event, theatre, live music performance)? |  |  |  |
| Been to a museum, art gallery or heritage site? |  |  |  |
| Been to a library? |  |  |  |
| Been out to eat or drink at a café, restaurant, pub or bar? |  |  |  |
| Been to a shopping centre, or mall, apart from regular shopping for food and household items? |  |  |  |
| Been to some other place of entertainment (e.g., dance, club, bingo, casino) |  |  |  |
| Been on any other outdoor trips (including going to places of natural beauty, picnics, going for a drive or going to the beach)? |  |  |  |
| Been to a day centre or any support groups?  Been to a local community social group? |  |  |  |
| Attended a religious group/activity/service? |  |  |  |
| Visited friends |  |  |  |
| Been visited by friends |  |  |  |
| Other: (please specify) |  |  |  |
