# Supplementary material for: Participation in leisure activities and quality of life of people with psychosis in England: a multi-site cross-sectional study
Source: Ann Gen Psychiatry. 2023 Mar 13;22:8. doi: 10.1186/s12991-023-00438-1 (PMC10009983; doi:10.1186/s12991-023-00438-1)
Supplement: Supplementary file 2 — Additional file 2. Distribution Graphs. [file 12991_2023_438_MOESM2_ESM.docx]

Supplementary Distribution graph


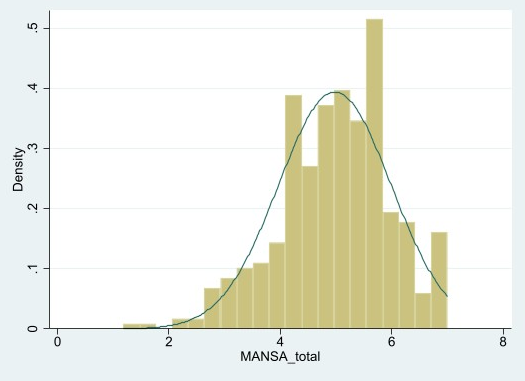


Quality of life MANSA score graph distribution


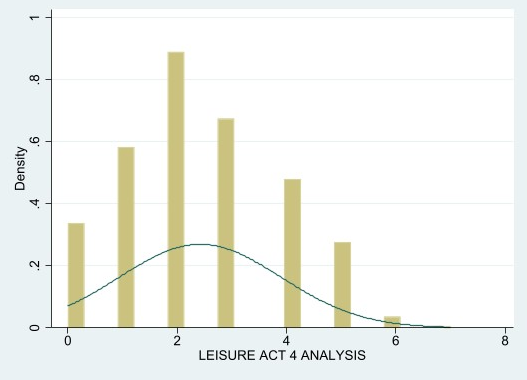


Leisure activities graph distribution


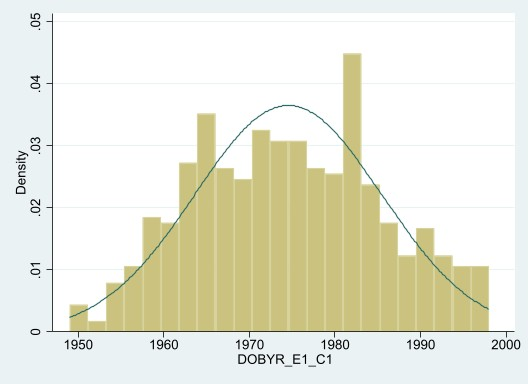


Age graph distribution


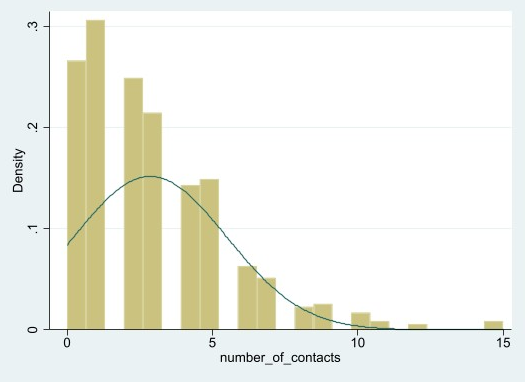


Social contacts graph distribution
